# Early Prognostic Factors for the Progress of Preeclampsia – Our Experience in the Period 2010-2011

**DOI:** 10.3889/oamjms.2016.065

**Published:** 2016-08-06

**Authors:** Mariya Angelova, Ivan Todorov, Emil Kovachev

**Affiliations:** 1 *Department of Obstetrics and Gynecology, Trakia University, Faculty of Medicine, Stara Zagora, Bulgaria*; 2 *Department of Obstetrics and Gynecology, Paraskev Stoyanov Medical University, Varna, Bulgaria*

**Keywords:** Preeclampsia, Doppler test, Pregnancy-associated plasma protein A, Body Mass Index

## Abstract

**AIM:**

To determine the prognostic value of the low Pregnancy-associated plasma protein A (PAPP-A) levels in the early stages of pregnancy (11–13 weeks GA) independently and in combination with a Doppler test of the uterine arteries during the second half of pregnancy (22–23 weeks GA).

**MATERIAL AND METHODS:**

The study covered the period 2010–2011 and included 106 pregnant women, aged 35–40, with a single child pregnancy. The research excluded pregnant women with anomalies of the fetus, smokers and women taking prophylactically low doses of aspirin.

**RESULTS:**

Thirty-six pregnant women had PAPP-A level below 0.4 MoM, whereas 20 of them developed preeclampsia and 7 – early preeclampsia. The combination of the low PAPP-A values and the abnormal Doppler test of the uterine arteries is with a considerably better prognostic value in regards to the risk of developing preeclampsia.

**CONCLUSION:**

The Doppler test is a non-invasive, quick and easy method for assessment of the uterine-placental blood flow.

## Introduction

Preeclampsia (PE) is one of the most serious complications during the second half of pregnancy [1]. The high perinatal maternal mortality and the children’s pathology make it a priority in the research of a number of scientists and researchers [2].

Our main goal was on the grounds of separate factors or a group of factors to determine who of the pregnant women display a high risk of developing preeclampsia with a view to the subsequent antenatal cares for them. In addition, we aimed to select markers that are comparatively easy, accessible and cost-effective for us and for our patients.

Our task was to determine the prognostic value of the low PAPP – A levels in the early stages of pregnancy (11 – 13 weeks GA) independently and in combination with a Doppler test of the uterine arteries during the second half of pregnancy (22 – 23 weeks GA).

## Material and Methods

The study covered the period 2010 – 2011 and included 106 pregnant women, aged 35 – 40, with a single child pregnancy. The research excluded pregnant women with anomalies of the fetus, smokers and women taking prophylactically low doses of aspirin.

Out of all pregnant women observed, 26 developed preeclampsia and 7 of them – early preeclampsia (before 34 weeks GA). In the beginning of the pregnancy, 27 of them were registered for increased Body Mass Index (BMI – 26 kg/m^2^). Thirteen out of all 26 women with preeclampsia had it during their previous pregnancies as well (Table 1).

**Table 1:** Investigated parameters in the control group and in the patients with early preeclampsia and preeclampsia

	Preeclampsia	Early preeclampsia before 34 week GA	Control group
Combination of increased PI, RI, presence of incisures and reduced PAPP – A	26 / 28%	7 / 7%	33 / 35%
Presence of incisures in the early diastolic phase	22 / 23%	6 / 6%	30 / 32%
Increased PI values	19 / 20%	6 / 6%	38 / 40%
Increased RI values	17 / 18%	5 / 5%	31 / 33%
Reduced levels of PAPP – A	14 / 15%	2 / 2%	36 / 38%
Anamnesis of previous preeclampsia	13 / 14%	4 / 4%	19 / 20%
Miscarriages in the anamnesis	5 / 5%	2 / 2%	29 / 31%
Increased BMI	12 / 13%	4 / 4%	27 / 29%
Increased RR values before pregnancy	27 / 29%	2 / 2%	32 / 34%
Accompanying diseases	6 / 6%	1 / 1%	22 / 23%

To examine the blood flow in the uterine arteries, MEDISON – AQUVIX V 10 and SONOACE X 8 devices were used. The blood flow velocity was registered by means of 3.5 or 5 MHz transabdominal sector array transducers. The test was performed in a standard manner, the patient lying down on her back, and a colour Doppler was used to chartering the uterine artery while a pulsating Power Doppler and a transducer, parallel to the uterine artery (from 15° – to 30°) were employed to determine pulsatility index (PI) and resistive index (RI).

The vascular resistance changes in the course of pregnancy. With its advance, the curves usually become low resistant – with low PI and RI and without diastolic incisures. The waveform was considered abnormal if there were bilateral notches with a mean RI of > 0.55, if there was a unilateral notch and a mean PI of > 0.65, or if the mean RI was 0.70.

**Figure 1:**
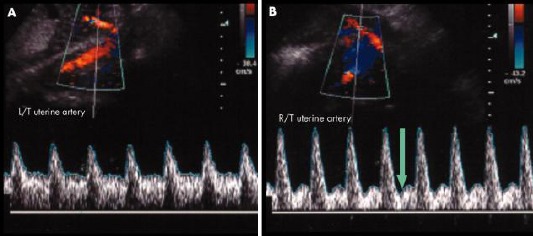
A) Normal blood flow; B) Abnormal blood flow

The quantitative measurement data of Pregnancy-associated plasma protein A (PAPP-A) were taken by the biochemical screening performed to these pregnant women at 11-13 week GA, employing the Delfia system for their laboratory determination.

The low PAPP-A levels in the first trimester of pregnant women without chromosome anomalies are connected with an unfavourable perinatal outcome – intrauterine growth retardation (IUGR), preeclampsia, miscarries. These namely were the pregnant women subject mandatory to a Doppler test of their umbilical artery. On the grounds of this survey, we drew the conclusion that women with increased PI of the umbilical artery develop more frequently IUGR. In the control group of 33 women with reduced PAPP-A, 16 pregnant women had IUGR.

The Doppler test of the two uterine arteries was also performed to all pregnant women in the control group.

## Results

The frequency of developing preeclampsia increases with the age of the mother with 35% which is above the average generally mentioned percentage.

To determine the prognostic value of PAPP-A in the first trimester, the following data have been collected: 36 pregnant women had PAPP-A level below 0.4 MoM, whereas 20 of them developed preeclampsia and 7 – early preeclampsia.

The combination of the low PAPP-A values and the abnormal Doppler test of the uterine arteries is with a considerably better prognostic value in regards to the risk of developing preeclampsia (Fig. 1).

**Figure 1:**
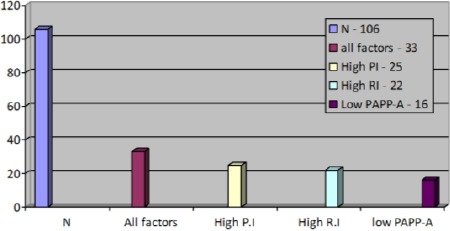
The combination of the low PAPP-A values and the abnormal Doppler test of the uterine arteries

## Discussion

The increased PI in both uterine arteries is a good and reliable prognostic indicator for preeclampsia. Besides, RI in one or in both arteries also plays a role [3]. Especially sensitive turned out the factor with which we observed subjectively the presence of incisures in the early diastolic phase.

Low serum PAPP-A levels are associated with a higher incidence of PE. Increased maternal serum PAPP-A levels have been observed in established PE [4-6]. A multicenter study of 8839 women demonstrated a significant relationship between PAPP-A levels at or below the 5th percentile and IUGR preterm delivery, PE, and stillbirth [7].

Alteration in plasma levels of angiogenic factors is more pronounced in early preeclampsia during clinical disease compared to late-onset preeclampsia [8-11]. Wikström et al. demonstrate 43 times higher median plasma concentrations of sFlt-1 in early onset compared to a 3-fold increase in late-onset preeclampsia and 21 times lower median plasma concentrations of PlGF in early onset disease compared to a 5-fold decrease in late-onset disease [8].

Although there are multiple potential biomarkers for PE their efficacy has been inconsistent and comparisons are difficult because of heterogeneity between different studies. Two papers with meta-analysis on biomarkers in preeclampsia were published recently (2015). Wu et al. found low predictive values using individual biomarkers which included a disintegrin and metalloprotease 12 (ADAM-12), inhibin-A, pregnancy-associated plasma protein A (PAPP-A), placental growth factor (PlGF) and placental protein 13 (PP-13) [12]. In the second meta-analysis the objective was to investigate the accuracy of serum biochemical markers (Pregnancy-Associated Plasma Protein-A (PAPP-A), human Chorionic Gonadotropin (hCG), Placental Growth Factor (PlGF), Placental Protein 13 (PP13) used in first trimester serum screening in predicting preeclampsia, small for gestational age (SGA) and preterm delivery [13]. The results showed low predictive accuracy overall. For preeclampsia, the best predictor was PlGF. The predictive value of serum markers for early preeclampsia was better than that of late preeclampsia. For SGA the best predictor was PP13. For preterm delivery, the best predictor was PP13 [13].

The predictive value of all these factors, when combined, is significantly enhanced.

In conclusion, the Doppler test is a non-invasive, quick and easy method for assessment of the uterine-placental blood flow.
